# PURA-Related Neurodevelopmental Disorder: A Comprehensive Clinical Review of Genetics, Phenotype, and Emerging Therapeutic Strategies

**DOI:** 10.7759/cureus.105181

**Published:** 2026-03-13

**Authors:** Sadaf Afshar, Wajiha Ahmed, Elif Özge Çelik, Sumayyah S Younis, Colleen Campbell, Yamuna Karthika Rao Potluri, Jaskaran Kaur, Deepak Swaroop Meda, Kopparti K Manjari, Arvin Khachikian, Jayalekshmi Leena, Manju Rai

**Affiliations:** 1 Internal Medicine, Medical University of Lublin, Lublin, POL; 2 Internal Medicine, Foundation University Medical College, Islamabad, PAK; 3 Internal Medicine, Ambulance Service, Van Provincial Health Directorate, Ministry of Health, Van, TUR; 4 Medicine, School of Medicine, New Vision University, Tbilisi, GEO; 5 Child and Adolescent Health, University of West Indies, Kingston, JAM; 6 Internal Medicine, Dr. NTR University of Health Sciences, Vijayawada, IND; 7 Internal Medicine, Samuel Merritt University, Oakland, USA; 8 Internal Medicine, St. George’s University, St. George’s, GRD; 9 Internal Medicine, Somervell Memorial CSI Medical College, Thiruvananthapuram, IND; 10 Biotechnology, Shri Venkateshwara University, Gajraula, IND

**Keywords:** epilepsy, gene therapy, haploinsufficiency, ketogenic diet, neurodevelopmental disorder, pura syndrome

## Abstract

PURA-related neurodevelopmental disorder (PURA-NDD) is an ultra-rare genetic condition caused by heterozygous pathogenic variants in the PURA gene, leading to haploinsufficiency of the Pur-α protein, a critical regulator of neuronal development and RNA trafficking. The disorder is characterized by neonatal hypotonia, global developmental delay, intellectual disability, severely impaired speech, epilepsy, feeding difficulties, respiratory dysfunction, and multisystem involvement. Clinical presentation is heterogeneous, with variability in seizure burden, motor milestones, autonomic dysfunction, endocrine abnormalities, and musculoskeletal complications such as scoliosis. Diagnosis relies on molecular confirmation through genome-wide sequencing, with early recognition enabling anticipatory guidance and multidisciplinary management. Current treatment remains supportive and symptom-directed, focusing on seizure control, nutritional support, respiratory care, rehabilitative therapies, and management of associated comorbidities. Evidence for targeted interventions, including ketogenic diet, vagus nerve stimulation, and neuromuscular junction-modulating agents, remains limited to case-based and small-cohort data. Emerging disease-modifying strategies, including adeno-associated virus-mediated gene replacement, antisense oligonucleotide approaches, RNA-targeted therapies, and drug repurposing, are under preclinical investigation. Advances in animal models, epigenetic profiling, and global patient registries are refining genotype-phenotype correlations and informing future clinical trial design. This comprehensive clinical review synthesizes current evidence on the genetics, pathophysiology, clinical spectrum, diagnostic evaluation, management strategies, and evolving therapeutic landscape of PURA-NDD, highlighting practical considerations for clinicians and priorities for translational research.

## Introduction and background

PURA-related neurodevelopmental disorder (PURA-NDD), commonly referred to as PURA syndrome, is an ultra-rare genetic condition that often first becomes apparent in early infancy. A typical clinical presentation may involve a newborn with severe hypotonia, feeding difficulties, and episodes of respiratory instability. As the child grows, global developmental delay becomes evident, and many affected individuals develop epilepsy and profound expressive language impairment, often remaining minimally verbal throughout life. Recognition of this constellation of features is important for clinicians evaluating infants and children with unexplained hypotonia and developmental delay.

PURA-NDD was first delineated in 2014 following the identification of de novo pathogenic variants in the PURA gene in individuals with severe neurodevelopmental delay and hypotonia [1-2]. The PURA gene, located on chromosome 5q31.3, encodes Pur-α, a highly conserved nucleic acid-binding protein that plays a critical role in neuronal proliferation, dendritic maturation, RNA transport, and regulation of gene transcription [3-4]. Loss of function of one copy of the gene results in haploinsufficiency, meaning that a single remaining functional gene copy cannot produce sufficient Pur-α protein to support normal neuronal development [5].

Since its initial description, the increasing availability of exome and genome sequencing has led to improved recognition of PURA-NDD worldwide. More than 700 affected individuals have been documented across multiple countries, with the vast majority of cases resulting from de novo heterozygous variants [6-7]. Although the estimated incidence is approximately one in 1,000,000 live births, the true prevalence is likely underestimated due to phenotypic variability and limited access to genetic testing in certain regions [7-8].

Clinically, PURA-NDD is characterized by neonatal hypotonia, global developmental delay, intellectual disability, markedly impaired or absent speech, epilepsy, feeding difficulties, respiratory dysregulation, and multisystem involvement [5,9-10]. The phenotype is heterogeneous, ranging from profound neurodevelopmental impairment to milder inherited presentations [11]. In addition to neurological manifestations, affected individuals may exhibit autonomic dysfunction, endocrine abnormalities, musculoskeletal complications such as scoliosis, and ocular findings [12-14]. This broad clinical spectrum often results in delayed diagnosis, particularly in individuals born before the widespread adoption of genomic sequencing technologies [15].

Advances in molecular characterization have expanded understanding of genotype-phenotype correlations, which refer to attempts to relate specific genetic variants to particular clinical features or disease severity [16-17]. Concurrently, emerging studies have identified epigenetic signatures, or characteristic patterns of DNA methylation associated with PURA variants, which may help clarify disease mechanisms and potentially assist with variant interpretation in the future [17]. Growing international collaboration, patient registries, and natural history studies are refining clinical expectations and identifying potential outcome measures for future interventional trials [6,18]. While current management remains primarily supportive and symptom-directed, emerging translational research, including gene replacement strategies, RNA-targeted therapies, and drug repurposing approaches, has generated cautious optimism for disease-modifying interventions [19-20].

Given the rarity of PURA-NDD and its complex multisystem presentation, clinicians may encounter challenges in diagnosis, anticipatory guidance, and long-term management. This comprehensive clinical review synthesizes current evidence on the genetics, pathophysiology, clinical manifestations, diagnostic evaluation, and management strategies of PURA-NDD, while also highlighting emerging therapeutic directions and priorities for translational research.

## Review

Methodology

This comprehensive clinical review was conducted using a structured literature search to identify relevant publications on PURA-NDD. Electronic databases, including PubMed/Medical Literature Analysis and Retrieval System Online (MEDLINE), Scopus, Web of Science, and Google Scholar, were searched for articles published between January 2014 (the year of the first clinical description of PURA syndrome) and September 2025.

Search terms included combinations of the following keywords and Medical Subject Headings (MeSH): “PURA syndrome,” “PURA-related neurodevelopmental disorder,” “PURA mutation,” “Pur-alpha,” “5q31.3 microdeletion,” “developmental delay,” “epilepsy,” “hypotonia,” “gene therapy,” “antisense oligonucleotides,” “RNA-targeted therapy,” “ketogenic diet,” and “vagus nerve stimulation.” Boolean operators (“AND,” “OR”) were used to refine the search strategy.

**Figure 1:**
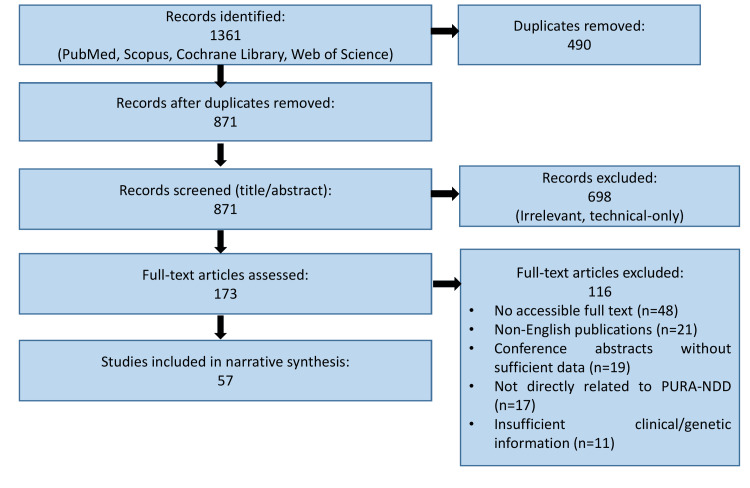
: Flow diagram summarizing the study selection process for the included literature in this comprehensive review. The structure of this diagram was adapted in accordance with the PRISMA 2020 framework [21]. This figure has been created by Sumayyah Sabah Younis using PowerPoint (Microsoft Corp., Redmond, WA, USA). PRISMA: neurodevelopmental disorder; NDD: neurodevelopmental disorder

Screening of studies was performed in multiple stages. Titles and abstracts were initially reviewed to identify potentially relevant studies, followed by full-text evaluation of eligible articles. During full-text assessment, 116 articles were excluded for the following reasons: lack of accessible full text (n = 48), non-English publications (n = 21), conference abstracts without sufficient methodological or clinical detail (n = 19), studies not directly related to PURA-related neurodevelopmental disorder (n = 17), and articles with insufficient clinical or genetic information (n = 11). The selection process emphasized studies contributing to the understanding of genetics, molecular mechanisms, clinical presentation, diagnostic strategies, management approaches, and emerging therapeutic developments. Due to the ultra-rare nature of PURA-NDD and the limited availability of large-scale trials, evidence synthesis was narrative rather than quantitative. Findings were thematically organized into clinical, genetic, translational, and management domains to provide a structured and clinically applicable overview. Figure 1 presents a flow diagram illustrating the literature identification, screening, eligibility assessment, and inclusion process for studies considered in this review. Although elements of the Preferred Reporting Items for Systematic Reviews and Meta-Analysis (PRISMA) framework [21] were applied to enhance transparency in literature identification and selection, this review was designed as a comprehensive synthesis rather than a formal systematic review and therefore does not include quantitative pooling or risk-of-bias assessment.

However, this review has several limitations. Several potentially relevant studies were excluded because full-text articles were unavailable or publications were not available in English. As a result, data from certain geographic regions and healthcare settings may be underrepresented. Consequently, the epidemiologic estimates, phenotypic spectrum, and management approaches summarized in this review may reflect a bias toward cohorts from centers with greater access to genomic testing and English-language publication. These limitations should be considered when interpreting the findings and generalizing conclusions to global populations affected by PURA-NDD.

Epidemiology and natural history

Incidence and Genetic Origin

PURA-NDD is classified as an ultra-rare condition, with an estimated incidence of approximately one in 1,000,000 live births [7-8]. Since its initial description in 2014, more than 700 cases have been reported globally, spanning over 60 countries [6-7]. However, the true prevalence is likely underestimated due to phenotypic variability, limited access to genomic testing in certain regions, and the possibility of milder or atypical presentations remaining undiagnosed [22-23].

The vast majority of reported cases arise from de novo heterozygous pathogenic variants in the PURA gene, accounting for approximately 99% of individuals diagnosed to date [7-8]. Inherited cases are rare but have been documented, including mild maternal transmission, highlighting the importance of parental testing and genetic counseling [11]. No founder mutations have been consistently identified, and pathogenic variants include missense, nonsense, frameshift, and small deletion changes, all contributing to functional haploinsufficiency [4].

Diagnostic Delay and Evolving Recognition

Diagnosis of PURA-NDD is frequently delayed due to nonspecific neonatal features and overlap with other hypotonic or developmental syndromes. Early manifestations, such as severe hypotonia, feeding difficulties, respiratory instability, and hypersomnolence, may resemble conditions including Prader-Willi syndrome or other neonatal encephalopathies [24]. Prior to the widespread implementation of exome sequencing and genome sequencing, many individuals underwent prolonged diagnostic evaluations without molecular confirmation.

Cohort analyses indicate that the average age at diagnosis is around seven years, although some individuals are not identified until adolescence or even adulthood [9]. The increasing use of genome-wide sequencing as a first- or second-tier test for children with developmental delay or congenital anomalies has significantly improved early detection [25]. Earlier diagnosis facilitates anticipatory management, targeted surveillance, and appropriate family counseling regarding recurrence risk [18].

Natural History and Clinical Trajectory

The natural history of PURA-NDD is characterized by significant phenotypic heterogeneity but consistent early neurodevelopmental impairment. Neonatal hypotonia is nearly universal and often accompanied by feeding difficulties and respiratory dysregulation [5,24]. Developmental delay becomes evident in infancy, with delayed acquisition of gross motor milestones and severely impaired expressive language development [9].

Large cohort analyses demonstrate that head control and independent sitting are substantially delayed compared with neurotypical peers, and only a minority of individuals achieve independent ambulation [9]. Speech outcomes are particularly limited, with most individuals remaining minimally verbal or nonverbal throughout life [9]. Epilepsy develops in approximately 50% to 60% of affected individuals and may present with diverse seizure types, sometimes evolving into developmental and epileptic encephalopathy [9-10].

Beyond early childhood, the clinical course may include progressive musculoskeletal complications such as scoliosis, contractures, and hip dysplasia, largely secondary to chronic hypotonia and motor impairment [12-13]. Autonomic dysfunction, sleep-related breathing abnormalities, endocrine disturbances, and ophthalmologic findings further contribute to long-term morbidity [5,12]. In rare cases, regression of previously acquired motor skills has been observed during adolescence or adulthood, underscoring the need for ongoing surveillance [26].

Although survival into adulthood is common, morbidity remains significant, primarily related to respiratory complications, refractory epilepsy, and feeding difficulties [27]. The limited availability of long-term prospective data highlights the need for structured natural history studies and global registries to better define lifespan outcomes and inform clinical trial readiness [6,16].

Genetics and pathophysiology

PURA Gene Biology

The PURA gene is located on chromosome 5q31.3 and encodes Pur-α, a highly conserved single-stranded DNA- and RNA-binding protein expressed widely in neural tissue [3-4]. Pur-α contains three conserved PUR domains (PUR I-III), which facilitate its interaction with purine-rich nucleic acid sequences and enable its role in DNA replication, transcriptional regulation, mRNA transport, and local translation within neurons [3].

In the nucleus, Pur-α participates in the regulation of gene transcription and cell cycle control, influencing neuronal differentiation and proliferation [3-4]. In the cytoplasm, it associates with RNA granules and contributes to dendritic mRNA trafficking, a process essential for synaptic maturation and plasticity [16]. These functions explain why reduced Pur-α expression disproportionately affects the developing central nervous system.

Haploinsufficiency of the PURA gene is considered the principal pathogenic mechanism underlying PURA-NDD [28]. This mechanism is supported by both human genetic data and animal model studies.

Variant Spectrum

Most individuals with PURA-NDD harbor de novo heterozygous pathogenic variants, including missense mutations, nonsense variants, frameshift changes, and small deletions [9,29]. Approximately 10% of cases are associated with nonrecurrent 5q31.3 microdeletions encompassing the PURA locus [18]. Inherited variants are rare but have been reported, often associated with milder phenotypes, emphasizing the need for parental testing and careful genetic counseling [11]. 

Most individuals with PURA-related neurodevelopmental disorder harbor de novo heterozygous pathogenic variants, including missense mutations, nonsense variants, frameshift changes, and small insertions or deletions that disrupt normal Pur-α function [9,29]. Approximately 10% of cases are associated with nonrecurrent 5q31.3 microdeletions encompassing the PURA locus [18]. Pathogenic variants have been reported throughout the coding region of the gene without a clear mutational hotspot, although several recurrent variants affecting functionally important domains of the Pur-α protein have been described in multiple cohorts [7,16].

Inherited variants are uncommon but have been documented, often in the context of milder neurodevelopmental phenotypes or parental mosaicism, emphasizing the importance of parental testing and careful genetic counseling [11]. Increasing numbers of cases have been reported from diverse geographic regions, including Europe, North America, and Asia, reflecting the expanding use of genomic sequencing in the evaluation of developmental delay and epilepsy [7-8]. Despite these advances, current data suggest that many cases remain underdiagnosed, particularly in regions with limited access to genomic testing.

Although more than 300 unique pathogenic variants have been described, genotype-phenotype correlations remain modest [7,16]. Earlier cohort studies did not identify strong associations between variant type or location and clinical severity [5,9]. More recent analyses suggest that truncating variants may be associated with more severe speech impairment, but overall predictive value remains limited [16]. Thus, clinical heterogeneity cannot be reliably inferred from genotype alone.

Epigenetic and Mechanistic Insights

Emerging evidence suggests that PURA-NDD may be associated with a shared epigenetic signature. Genome-wide methylation profiling has identified a distinct DNA methylation pattern in affected individuals, independent of variant subtype, further supporting a unified haploinsufficiency mechanism [17]. This epigenetic signature may eventually serve as a diagnostic biomarker or a tool for monitoring therapeutic response.

Animal models provide additional insight into disease mechanisms. Homozygous Pur-α-deficient mice exhibit severe neurological abnormalities, including tremors, seizures, impaired neuronal maturation, megalencephaly, and early lethality [30-31]. Heterozygous mice, which more closely approximate the human condition, demonstrate motor impairment, memory deficits, reduced dendritic complexity, and neuronal loss in the hippocampus and cerebellum [32]. These findings reinforce the central role of Pur-α in neuronal proliferation, dendritic architecture, and synaptic integrity.

Zebrafish and Drosophila models have further illustrated the importance of Pur-α in neural development and RNA homeostasis, though species differences limit direct translational extrapolation [33-37]. Notably, Pur-α has also been implicated in RNA toxicity pathways in repeat expansion disorders, highlighting its broader biological relevance in neurodegenerative processes [34-36]. While PURA-NDD is not a classic repeat expansion disorder, these findings underscore the importance of RNA regulation in disease pathogenesis.

Clinical-Translational Implications

Understanding the molecular basis of PURA-NDD has direct implications for therapeutic development. Because haploinsufficiency drives disease, strategies aimed at restoring or augmenting Pur-α expression, such as adeno-associated virus (AAV)-mediated gene replacement, RNA-targeted therapies, or modulation of regulatory elements, represent rational approaches [20]. However, given Pur-α’s involvement in multiple cellular pathways and its association with oncogenic processes when overexpressed, careful dose optimization and tissue-specific targeting will be essential in translational applications [38].

**Figure 2:**
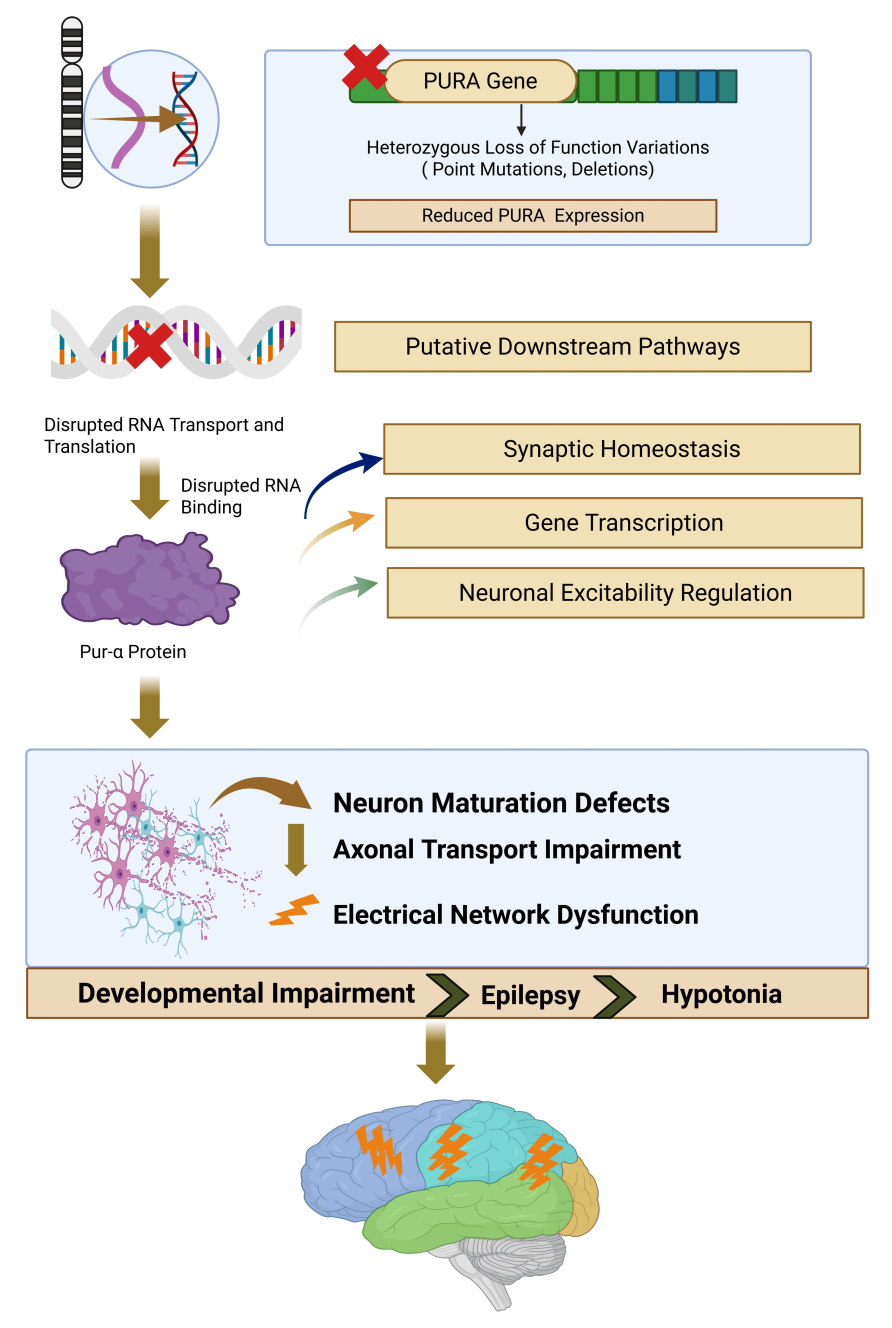
Genetics and pathophysiology of PURA-related neurodevelopmental disorder Heterozygous loss-of-function variants in the PURA gene on chromosome 5q31.3 lead to reduced Pur-α expression and impaired RNA binding, disrupting neuronal maturation, synaptic homeostasis, and network excitability, ultimately resulting in developmental impairment, epilepsy, and hypotonia. This figure has been created by Jaskaran Kaur using Biorender.com.

Collectively, current genetic and mechanistic data support PURA-NDD as a disorder of impaired neuronal maturation and RNA regulation, providing a biologically coherent framework for future disease-modifying interventions. The proposed molecular cascade linking PURA haploinsufficiency to neuronal dysfunction and clinical manifestations is illustrated in Figure 2.

Clinical manifestations

PURA-NDD is a multisystem condition with predominant neurological involvement beginning in the neonatal period. Although the phenotype is heterogeneous, several core features, including neonatal hypotonia, severe expressive language impairment, epilepsy, feeding difficulties, and respiratory abnormalities, are consistently reported across published cohorts. 

Neuromuscular and Neurological Manifestations

Severe neonatal hypotonia is the hallmark presenting feature of PURA-NDD and has been reported in nearly all affected individuals in multiple cohorts, including studies of more than 30 genetically confirmed patients [5,24]. Hypotonia frequently leads to poor feeding, weak suck, diminished spontaneous movements, and respiratory instability, including hypoventilation or apnea. Many neonates require supportive respiratory measures or feeding assistance during the early postnatal period [10,24].

Global developmental delay becomes evident in infancy. Gross motor milestones are significantly delayed, with delayed head control, independent sitting, and ambulation compared with neurotypical peers [9]. A substantial proportion of individuals do not achieve independent walking. Expressive language impairment is particularly severe; in cohort analyses involving over 50 individuals with PURA-NDD, more than 90% were reported to be minimally verbal or nonverbal throughout life [9].

Epilepsy occurs in approximately 50% to 60% of affected individuals, as reported in cohort studies including more than 60 genetically confirmed patients, and represents a major contributor to morbidity [9-10]. Seizure onset typically occurs in infancy or early childhood and may include focal, generalized, or multifocal seizure types. Some individuals develop developmental and epileptic encephalopathy [9]. Electroencephalography (EEG) findings are variable and may demonstrate generalized or focal epileptiform discharges [19,39]. Brain magnetic resonance imaging (MRI) commonly reveals nonspecific abnormalities, including delayed myelination, thinning of the corpus callosum, cerebral atrophy, and ventricular prominence [10,12,40].

Movement disorders such as chorea, dystonia, ataxia, and myoclonus have been reported, particularly in older children and adolescents [13]. In rare cases, regression of previously acquired motor skills has been observed during adolescence or adulthood [9].

Autonomic and Respiratory Features

Autonomic dysfunction is frequently reported and may manifest as hypersomnolence, hypothermia, constipation, and excessive intrauterine or postnatal hiccups [5]. Sleep-related breathing abnormalities, including central and obstructive sleep apnea, are common and may require oxygen supplementation or noninvasive ventilation [2,9].

Respiratory complications represent a significant source of morbidity and, in severe cases, mortality. Neonatal respiratory dysregulation may improve over time; however, chronic vulnerability to respiratory compromise persists in some individuals [9-10].

Feeding and Gastrointestinal Manifestations

Feeding difficulties are nearly universal in early life and are largely attributable to hypotonia and impaired coordination of swallowing [5,24]. Gastroesophageal reflux, dysphagia, drooling (sialorrhea), and constipation are frequently observed. Some individuals require nasogastric or gastrostomy tube placement to ensure adequate nutrition and reduce aspiration risk [41-42].

Chronic feeding impairment may contribute to growth failure, recurrent respiratory infections, and reduced quality of life for both patients and caregivers.

Musculoskeletal and Orthopedic Complications

Chronic axial hypotonia and motor impairment predispose individuals to progressive musculoskeletal complications. Scoliosis is commonly reported and may progress during adolescence [12-13]. Hip dysplasia, contractures, and delayed bone mineralization may also occur. These complications often necessitate orthopedic surveillance and, in some cases, surgical intervention.

Facial and Ophthalmologic Findings

Facial dysmorphism is variably present and may include myopathic facies with an open mouth posture, high anterior hairline, tented upper lip, rounded cheeks, and high-arched palate [5,43]. Ocular findings such as strabismus, nystagmus, and cortical visual impairment are also reported [5,12]. However, facial features are not sufficiently specific to establish a clinical diagnosis without molecular confirmation.

Endocrine and Systemic Features

Endocrine abnormalities have been described in a subset of individuals, including vitamin D deficiency and abnormalities in thyroid, cortisol, or androgen levels [5]. The true prevalence remains uncertain due to inconsistent endocrine screening across cohorts.

Cardiac and urogenital anomalies are uncommon but have been reported, particularly in individuals with larger 5q31.3 deletions [18,40]. These findings support phenotype-guided systemic evaluation at diagnosis.

Behavioral and Cognitive Profile

Intellectual disability ranges from moderate to profound in most individuals with PURA-NDD [5,9]. Behavioral features may include irritability, poor sleep regulation, and features overlapping with autism spectrum disorder, although systematic behavioral characterization remains limited. Nonverbal communication methods and augmentative communication strategies are often required to support functional interaction.

Taken together, PURA-NDD is characterized by early-onset hypotonia, severe expressive language impairment, epilepsy in approximately half of affected individuals, and multisystem involvement affecting respiratory, gastrointestinal, musculoskeletal, and autonomic domains. The breadth of manifestations underscores the need for coordinated multidisciplinary care and anticipatory monitoring across the lifespan. The major clinical manifestations and their approximate frequencies are summarized in Table 1.

**Table 1:** Clinical spectrum of PURA-related neurodevelopmental disorder

System/Domain	Key Clinical Features	Number of Patients Reported in Key Cohorts	Approximate Frequency	Clinical Notes / Diagnostic Clues	Key Differential Diagnoses	Key References
Neuromuscular	Severe neonatal hypotonia	~95 patients	>90–95%	Often presenting feature; contributes to feeding and respiratory difficulties	Prader–Willi syndrome, congenital myopathies, and spinal muscular atrophy	[5,9,24]
	Delayed gross motor milestones	~60–70 patients	>90%	Delayed head control, sitting, and ambulation	Hypotonic cerebral palsy, mitochondrial disorders	[9]
	Limited or absent independent walking	~60 patients	~60%	Persistent motor impairment common	Congenital myopathy, Rett syndrome	[9]
Speech & Cognitive	Global developmental delay	>100 reported cases	Nearly universal	Evident in infancy	Rett syndrome, Angelman syndrome	[5,9]
	Moderate to profound intellectual disability	~60–80 patients	>80%	Requires lifelong support	Fragile X syndrome, Angelman syndrome	[5,9]
	Severe expressive language impairment	~60 patients	>90% minimally verbal/nonverbal	Hallmark feature	Rett syndrome, FOXP1/FOXP2-related disorders	[9,12]
Epilepsy	Focal, generalized, or multifocal seizures	~60 patients	50–60%	Onset typically in infancy or early childhood	Dravet syndrome, Lennox–Gastaut syndrome	[9-10]
	Developmental and epileptic encephalopathy	Subset	~15–20%	Associated with severe neurological impairment	SCN2A-related epilepsy, CDKL5 deficiency disorder	[9,39]
Neuroimaging Findings	Delayed myelination	~40 patients	40–50%	Nonspecific MRI finding	Leukodystrophies, mitochondrial disorders	[10,12,40]
	Corpus callosum thinning	~30–40 patients	30–40%	Supportive but not diagnostic	Aicardi syndrome, agenesis of corpus callosum syndromes	[10,12]
	Cerebral atrophy / ventricular prominence	~30 patients	20–30%	Exclude alternative etiologies	Neurodegenerative disorders	[2,10]
Movement Disorders	Chorea, dystonia, ataxia, myoclonus	~20 patients	20–30%	More common in older children	ADCY5-related dyskinesia, inherited dystonia syndromes	[13]
Respiratory	Neonatal hypoventilation/apnea	~40–50 patients	40–50%	May improve with age	Congenital central hypoventilation syndrome	[10,24]
	Sleep apnea (central/obstructive)	~30 patients	~30–40%	Consider polysomnography	Obstructive sleep apnea, Prader–Willi syndrome	[2,9]
Autonomic	Hypersomnolence	~40 patients	50–60%	Often early-life feature	Narcolepsy, Prader–Willi syndrome	[5,24]
	Hypothermia	~15–20 patients	20–25%	Neonatal or infantile	Hypothalamic dysfunction syndromes	[5]
	Constipation	~40–50 patients	50–60%	May persist into childhood	Neuromuscular gastrointestinal dysmotility	[5,12]
Gastrointestinal	Feeding difficulties	~80–90 patients	>80–90%	Often requires feeding support	Prader–Willi syndrome, congenital myopathies	[5,24]
	Gastroesophageal reflux	~40–50 patients	50–60%	Related to hypotonia	Neuromuscular disorders	[5]
	Sialorrhea	~40 patients	40–50%	Impacts quality of life	Cerebral palsy	[44-45]
Musculoskeletal	Scoliosis	~40–50 patients	40–50%	Often progressive during adolescence	Neuromuscular scoliosis	[12-13]
	Hip dysplasia/contractures	~20–30 patients	20–30%	Secondary to chronic hypotonia	Congenital myopathies	[12-13]
Ophthalmologic	Strabismus	~30–40 patients	30–40%	May require correction	Congenital ocular motor disorders	[5,12]
	Nystagmus / cortical visual impairment	~20–30 patients	20–30%	Contributes to developmental delay	Optic nerve hypoplasia syndromes	[12]
Endocrine	Vitamin D deficiency	~20 patients	20–30%	Screening recommended in some cohorts	Nutritional deficiency disorders	[5]
	Thyroid/cortisol abnormalities	Few reported cases	<10–15%	Not routinely screened	Congenital endocrine disorders	[5]
Cardiac / Urogenital	Congenital anomalies	Limited cases	<10–15%	More frequent in 5q31.3 deletions	Chromosomal microdeletion syndromes	[18,40]

Diagnostic approach

When to Suspect PURA-NDD

Early recognition of PURA-NDD is essential to facilitate anticipatory management, coordinated multidisciplinary care, and appropriate genetic counseling. Because the clinical presentation overlaps with several neonatal hypotonic and developmental syndromes, molecular confirmation remains the cornerstone of diagnosis.

Clinical suspicion should arise in neonates or infants presenting with severe generalized hypotonia, feeding difficulties requiring medical support, neonatal hypoventilation or apnea, hypersomnolence, hypothermia, early-onset seizures, or profound expressive language delay. These features, particularly when occurring in combination, warrant early genomic evaluation [5,9,24]. Although neonatal respiratory instability may improve during infancy, global developmental delay and marked speech impairment typically become more evident over time [9-10]. In many cases, the onset of epilepsy prompts a neurological evaluation that ultimately leads to genetic testing [9].

Recommended Genetic Testing

Definitive diagnosis requires identification of a heterozygous pathogenic variant in the PURA gene or detection of a nonrecurrent 5q31.3 deletion encompassing the PURA locus [18]. Approximately 90% of affected individuals harbor sequence variants, while about 10% have microdeletions involving the gene [18]. Current recommendations from the American College of Medical Genetics and Genomics (ACMG) support the use of exome sequencing or genome sequencing as first- or second-tier diagnostic tests in children with congenital anomalies, developmental delay, or intellectual disability [25]. In the context of suspected PURA-NDD, genome-wide sequencing is preferred because of its higher diagnostic yield and its ability to detect both single-nucleotide variants and copy number variations [25,46].

Chromosomal microarray analysis remains useful for detecting 5q31.3 deletions and may be considered when a microdeletion is suspected [18,40]. Multigene panels for neurodevelopmental disorders or epilepsy may include PURA but can miss atypical presentations or novel variants [5]. Parental testing is recommended following identification of a pathogenic variant to determine recurrence risk and to detect rare inherited cases [11,18]. For unaffected parents, recurrence risk is generally low, estimated at less than 1% due to possible germline mosaicism; however, if a parent carries the pathogenic variant, the recurrence risk for future offspring is 50% [11,18].

Baseline Systemic Evaluation

After molecular confirmation, a structured baseline evaluation should be performed to assess disease severity and identify associated comorbidities. Neurological evaluation includes electroencephalography, particularly in individuals with seizures or developmental regression, as EEG abnormalities may be present even in the absence of clinically apparent seizures [9-10]. Brain magnetic resonance imaging may reveal supportive but nonspecific findings such as delayed myelination, thinning of the corpus callosum, cerebral atrophy, or ventricular prominence, and is useful in excluding alternative diagnoses [2,10,40].

Given the high prevalence of feeding difficulties and aspiration risk, formal swallowing assessment should be considered when clinically indicated. Video fluoroscopic swallow study or fiberoptic endoscopic evaluation of swallowing may be used depending on availability and clinical scenario [47-48]. Respiratory evaluation is also important, and polysomnography may be considered in individuals with persistent respiratory symptoms or suspected sleep-disordered breathing [2,9].

Systemic screening should be guided by clinical findings. Echocardiography may be appropriate when cardiac anomalies are suspected, particularly in individuals with larger chromosomal deletions [18,40]. Renal or urogenital ultrasound may be considered in selected cases [18]. Endocrine evaluation, including thyroid function testing, cortisol assessment, and vitamin D levels, may be performed when clinically indicated, although routine universal screening is not yet standardized [5].

The differential diagnosis of PURA-NDD includes other genetic and metabolic disorders presenting with neonatal hypotonia and developmental delay, such as Prader-Willi syndrome, congenital myopathies, mitochondrial disorders, and other developmental and epileptic encephalopathies [24]. Because the facial phenotype is variable and not pathognomonic, molecular testing is essential for confirmation.

**Figure 3:**
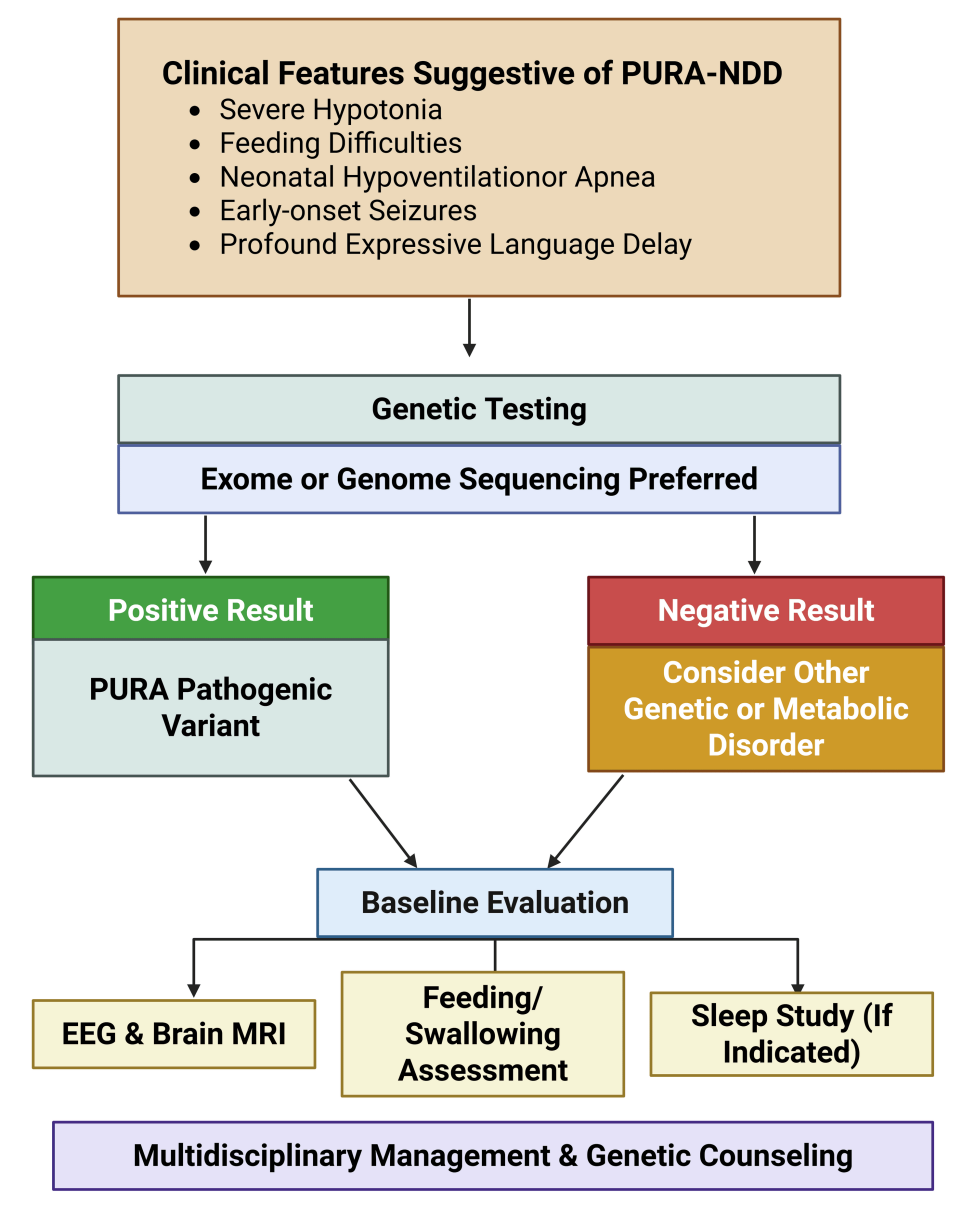
Diagnostic algorithm for PURA-related neurodevelopmental disorder (PURA-NDD) This figure has been created by Colleen Campbell using Biorender.com. EEG: electroencephalography; MRI: magnetic resonance imaging

Maintaining a low threshold for genomic testing in infants with unexplained hypotonia and global developmental delay is critical. Early diagnosis enables timely multidisciplinary intervention, informed prognostic counseling, and potential enrollment in registries and research initiatives that may facilitate access to future therapeutic developments [6,18]. Figure 3 explains the diagnostic algorithm of PURA-NDD.

Current standard management

Currently, treatment remains primarily supportive and symptom-directed. Management is therefore primarily supportive and symptom-directed, with emphasis on early multidisciplinary involvement to reduce complications and optimize functional outcomes [18,44]. Care typically requires coordination among pediatricians, neurologists, clinical geneticists, rehabilitation specialists, gastroenterologists, pulmonologists, and orthopedic surgeons, depending on individual presentation.

Neurological management focuses primarily on seizure control and developmental support. Epilepsy occurs in approximately half of affected individuals and may present with diverse seizure types [9-10]. Antiseizure medications are selected based on seizure semiology and electroencephalographic findings, although no specific agent has demonstrated superiority in PURA-NDD. Seizures may be refractory in some individuals, necessitating combination therapy and long-term follow-up [9]. Ongoing neurological surveillance is essential, particularly in those with developmental regression or evolving seizure patterns.

Hypotonia is a nearly universal feature and significantly impacts motor development and respiratory function. Early referral to physical and occupational therapy is recommended to promote motor skill acquisition, prevent contractures, and improve functional mobility [49-50]. Therapy approaches are typically task-oriented and individualized, incorporating strengthening exercises, postural training, orthotic support when needed, and caregiver education. In selected cases, assistive mobility devices may enhance independence and quality of life.

Feeding difficulties represent a major source of morbidity in infancy and childhood. Impaired suck-swallow coordination and gastroesophageal reflux are common and may predispose to aspiration and recurrent respiratory infections [5,24]. Nutritional support may include feeding therapy, positioning strategies, thickened feeds, or, in more severe cases, nasogastric or gastrostomy tube placement [41-42]. Early intervention can reduce growth failure and improve respiratory outcomes.

Respiratory care is particularly important in neonates and individuals with sleep-disordered breathing. Neonatal hypoventilation may require temporary oxygen supplementation or ventilatory support [9-10]. In older children, evaluation for central or obstructive sleep apnea may be warranted, and noninvasive ventilation such as continuous positive airway pressure may be beneficial when indicated [2]. Preventive strategies to reduce respiratory infections, including vaccination and aspiration precautions, are also critical.

Sialorrhea can significantly impair quality of life and increase aspiration risk. Management options include behavioral strategies when feasible, pharmacologic therapy such as anticholinergic agents, and botulinum toxin injections to the salivary glands in selected cases [44-45]. Surgical approaches may be considered in refractory cases, although data specific to PURA-NDD remain limited.

Musculoskeletal complications such as scoliosis, hip dysplasia, and contractures require regular orthopedic surveillance. Progressive scoliosis may necessitate bracing or surgical correction depending on severity and functional impact [12-13]. Early rehabilitation plays a preventive role in limiting secondary deformities.

Perioperative management requires special consideration. Individuals with PURA-NDD may have an increased risk of difficult airway management due to hypotonia and craniofacial features, as well as heightened sensitivity to medications that depress respiratory drive [51]. Careful anesthetic planning and postoperative monitoring are therefore recommended.

Beyond medical management, psychosocial and developmental support are integral components of care. Most individuals require long-term speech therapy and alternative or augmentative communication strategies due to severe expressive language impairment [9]. Families benefit from genetic counseling, education regarding prognosis, and connection to patient support networks and registries [6,18]. Transition planning for adolescence and adulthood should address educational, vocational, and medical continuity needs.

Overall, management of PURA-NDD requires lifelong surveillance and coordinated care tailored to individual clinical manifestations. Although current treatment remains supportive, early and proactive intervention can meaningfully improve quality of life and reduce secondary complications.

Symptom-targeted interventions with emerging evidence

In addition to standard supportive management, several symptom-targeted interventions have been explored in individuals with PURA-NDD. Most available data derive from case reports, small cohorts, or extrapolation from related neurological conditions. Therefore, these interventions should be considered on an individualized basis, with careful assessment of potential benefits and risks.

Seizure management remains a major therapeutic focus. Antiseizure medications are selected according to seizure type and electroencephalographic findings, as no single agent has demonstrated clear superiority in PURA-NDD [9-10]. Case reports describe the use of agents such as valproate, phenobarbital, nitrazepam, and clobazam, with variable long-term seizure control [42]. In some individuals, seizures may become more difficult to control over time, requiring dose escalation or combination therapy. Because epilepsy can significantly affect neurodevelopmental outcomes, close neurological follow-up is recommended.

The ketogenic diet has been explored in individuals with drug-resistant epilepsy associated with PURA-NDD. Case-based evidence suggests potential benefits in seizure reduction and, in some reports, improvement in sialorrhea [19]. However, adverse effects, including weakness and fatigue, have been described, and response appears variable [19]. Although ketogenic therapy is well established in certain metabolic and epileptic disorders, its role in PURA-NDD remains based on limited observational data and should be implemented under specialized supervision.

Vagus nerve stimulation has also been used in selected individuals with refractory epilepsy. Evidence from broader epilepsy populations demonstrates that vagus nerve stimulation may reduce seizure frequency and severity, although complete seizure freedom is uncommon [52-53]. Data specific to PURA-NDD are limited, but available reports suggest that vagus nerve stimulation may be considered in cases where pharmacologic therapy fails, and resective epilepsy surgery is not feasible. Careful multidisciplinary evaluation is necessary before implantation.

Emerging observations suggest that neuromuscular junction dysfunction may contribute to weakness and fatigability in some individuals with PURA-NDD [44,54]. In selected cases, treatment with pyridostigmine or salbutamol has resulted in symptomatic improvement, particularly in patients demonstrating decremental responses on repetitive nerve stimulation [53]. These findings raise the possibility that a subset of patients may exhibit treatable neuromuscular junction involvement. However, evidence remains preliminary, and further systematic evaluation is required before routine implementation.

Management of sialorrhea remains challenging, particularly in individuals with significant neuromotor impairment. Pharmacologic therapies such as glycopyrrolate may reduce drooling but are often limited by anticholinergic adverse effects [45]. Botulinum toxin injection into the parotid and submandibular glands has shown effectiveness in pediatric sialorrhea of various etiologies, with temporary reduction in salivary flow [45,55]. Although not extensively studied in PURA-NDD specifically, this approach may be considered in refractory cases. Surgical interventions, including duct ligation or gland excision, are reserved for severe, persistent cases due to their invasive nature [56].

Sleep-disordered breathing may require targeted intervention when clinically significant. Noninvasive ventilation, such as continuous positive airway pressure, can improve oxygenation and reduce apnea episodes in individuals with central or obstructive sleep apnea [39]. Adenotonsillectomy may be considered in selected patients with obstructive pathology, although outcome data specific to PURA-NDD are limited [57].

Orthopedic interventions may be necessary for progressive scoliosis or joint contractures. Surgical correction has been reported in individual cases with functional improvement, but perioperative risks must be carefully weighed, given respiratory vulnerability and hypotonia [51,53].

Overall, symptom-targeted interventions in PURA-NDD are guided by extrapolation from related neurological conditions and limited disorder-specific evidence. Individualized decision-making, multidisciplinary evaluation, and careful monitoring remain essential. Larger cohort studies and prospective trials are needed to clarify the efficacy and safety of these interventions within this rare population.

Emerging disease-modifying strategies

Advances in molecular genetics and translational neuroscience have prompted exploration of potential disease-modifying strategies for PURA-NDD. Given that haploinsufficiency of the PURA gene is the principal pathogenic mechanism, therapeutic approaches aimed at restoring or augmenting Pur-α expression represent a rational direction for intervention [5,16].

Gene replacement therapy using AAV vectors is one of the most conceptually direct approaches. AAV-mediated delivery of a functional PURA gene copy could theoretically restore protein levels in affected neurons. Preclinical gene therapy platforms have demonstrated feasibility in other monogenic neurological disorders, supporting this strategy in principle [20]. However, several challenges must be addressed before clinical application. These include optimal vector design, achieving adequate distribution across relevant brain regions, ensuring long-term expression, and avoiding excessive Pur-α overexpression. Notably, Pur-α has been implicated in oncogenic pathways when dysregulated, underscoring the importance of controlled and tissue-specific expression [58]. At present, no human trials of AAV-based therapy for PURA-NDD have been initiated.

RNA-targeted approaches represent another promising avenue. Antisense oligonucleotides (ASOs) have demonstrated therapeutic success in other neurological disorders by modulating RNA stability or translation. In the context of PURA-NDD, theoretical strategies include enhancing transcription from the intact allele, stabilizing PURA messenger RNA, or modifying regulatory elements to increase protein expression. Although these approaches remain preclinical, advances in RNA therapeutics and delivery systems provide a framework for future development [20]. Epigenetic profiling studies identifying a shared DNA methylation signature in affected individuals further suggest that molecular biomarkers could eventually guide therapeutic monitoring [17].

Drug repurposing offers a pragmatic and potentially accelerated pathway toward therapeutic intervention. Existing medications with known safety profiles may be evaluated for their ability to modify downstream pathways affected by Pur-α deficiency. Observations of neuromuscular junction dysfunction in a subset of individuals have prompted exploratory use of agents such as pyridostigmine and salbutamol, with reported symptomatic improvement in select cases [44,54]. While these findings do not constitute disease modification, they highlight mechanistically informed treatment strategies and underscore the importance of phenotype-driven therapeutic exploration.

Animal models continue to play a critical role in therapeutic development. Murine models of Pur-α deficiency demonstrate impaired neuronal maturation, motor dysfunction, and seizure susceptibility, supporting the biological plausibility of gene restoration approaches [30-32]. Zebrafish and Drosophila systems provide complementary platforms for mechanistic studies and high-throughput drug screening, although interspecies differences limit direct translational extrapolation [33-37]. Refinement of heterozygous models that more closely mimic the human condition will be essential for preclinical testing.

Progress toward disease-modifying therapy in PURA-NDD will depend on integration of molecular insights, robust preclinical validation, and collaborative infrastructure. Natural history studies and international registries are critical to defining clinical endpoints, stratifying phenotypic variability, and establishing trial readiness [6,18]. Given the rarity of the disorder, multicenter collaboration will be essential to achieve sufficient statistical power for future clinical trials.

At present, disease-modifying treatment remains investigational. However, the growing understanding of PURA biology, combined with advances in gene and RNA therapeutics, provides a biologically coherent foundation for future targeted interventions.

Current and emerging therapeutic approaches in PURA-NDD are summarized in Table 2.

**Table 2:** Current and emerging therapeutic strategies in PURA-related neurodevelopmental disorder AAC: augmentative and alternative communication; CPAP: continuous positive airway pressure; NMJ: neuromuscular junction; AAV: adeno-associated virus

Therapeutic Category	Intervention	Target Domain	Evidence Level	Key References	Clinical Status
Supportive Standard Care	Antiseizure medications	Epilepsy	Cohort data, case series	[9-10]	Standard of care
	Physical and occupational therapy	Hypotonia, motor delay	Systematic reviews (developmental hypotonia)	[49-50]	Standard of care
	Speech therapy and AAC	Severe expressive impairment	Cohort data	[9]	Standard of care
	Nutritional support/gastrostomy	Feeding dysfunction	Case series	[41-42]	Standard of care
	Noninvasive ventilation (CPAP)	Sleep-disordered breathing	Observational data	[2,9]	Standard of care (when indicated)
Symptom-Targeted (Emerging Evidence)	Ketogenic diet	Drug-resistant epilepsy	Case reports, small cohorts	[19]	Selective use
	Vagus nerve stimulation	Refractory epilepsy	Extrapolated epilepsy data	[52-53]	Consider in selected cases
	Pyridostigmine/salbutamol	Suspected NMJ dysfunction	Case reports	[44,54]	Investigational / phenotype-driven
	Botulinum toxin (salivary glands)	Sialorrhea	Pediatric sialorrhea data	[45,55]	Selective use
Investigational Disease-Modifying	AAV-mediated gene replacement	PURA haploinsufficiency	Preclinical	[20]	Experimental
	Antisense oligonucleotides	RNA modulation	Preclinical/theoretical	[20]	Experimental
	Epigenetic biomarker-guided therapy	Methylation signature	Translational research	[17]	Exploratory
	Drug repurposing strategies	Downstream pathway modulation	Conceptual/case-based	[44,54]	Early-stage exploration

Clinical research infrastructure and patient-centered priorities 

The rarity and phenotypic heterogeneity of PURA-NDD necessitate coordinated international research efforts to advance understanding and therapeutic development. Over the past decade, the establishment of patient registries, natural history studies, and collaborative research networks has significantly enhanced characterization of the disorder and facilitated data sharing across institutions [6,18].

Global patient registries, including those supported by advocacy organizations, serve as centralized repositories for clinical, genetic, and longitudinal data. These registries help define the full phenotypic spectrum of PURA-NDD, clarify genotype-phenotype associations, and identify potential clinical endpoints relevant to interventional trials [6]. Structured data collection also enables estimation of disease prevalence, evaluation of long-term outcomes, and recognition of rare complications that may not be apparent in isolated case reports.

Natural history studies are particularly critical in rare neurodevelopmental disorders. Because disease-modifying therapies are not yet available, prospective longitudinal data are essential to establish baseline rates of developmental progression, seizure burden, motor function decline, and respiratory morbidity [18]. Such information is indispensable for clinical trial design, including the determination of meaningful outcome measures and appropriate age windows for intervention.

Collaborative research networks linking clinicians, geneticists, neuroscientists, and patient advocacy groups facilitate multicenter studies and harmonization of clinical protocols. Given the limited number of affected individuals at any single institution, multicenter collaboration is required to achieve adequate sample sizes for observational studies and future therapeutic trials. Integration of standardized assessments across centers can improve data comparability and accelerate translational progress.

Patient-centered priorities play a central role in shaping research direction. Families frequently identify seizure control, communication ability, motor independence, feeding safety, and respiratory stability as key determinants of quality of life. Incorporating caregiver-reported outcomes and functional measures into study design ensures that research efforts align with real-world needs. In rare disorders such as PURA-NDD, meaningful clinical improvement may not require complete disease reversal but rather incremental gains in functional domains that substantially reduce caregiver burden and enhance daily living.

Ethical considerations are particularly important in the context of emerging gene- and RNA-based therapies. Transparent communication regarding potential risks, realistic expectations, and long-term monitoring requirements is essential. Engagement of patient advocacy organizations in trial planning can improve recruitment, adherence, and dissemination of findings.

Overall, strengthening clinical research infrastructure and prioritizing patient-centered outcomes are essential steps toward advancing therapeutic development in PURA-NDD. Continued collaboration among researchers, clinicians, and families will be fundamental to translating molecular insights into clinically meaningful interventions.

Practical recommendations

Management of PURA-NDD requires a proactive, multidisciplinary approach tailored to individual clinical manifestations. Because the disorder affects multiple organ systems and evolves over time, early recognition, structured surveillance, and anticipatory guidance are essential components of care.

Certain clinical features may serve as early diagnostic “red flags” that should prompt consideration of PURA-related neurodevelopmental disorder and early genomic evaluation. These include severe neonatal hypotonia accompanied by feeding difficulties, respiratory instability or hypoventilation in the neonatal period, hypersomnolence, and early developmental delay. Profound expressive language impairment that appears disproportionate to other developmental domains, particularly when associated with epilepsy or abnormal electroencephalographic findings, should further raise clinical suspicion. Additional clues include persistent feeding dysfunction requiring nutritional support, recurrent respiratory complications, autonomic abnormalities such as hypothermia or constipation, and progressive musculoskeletal complications, including scoliosis. When several of these features occur together, especially in the absence of an alternative diagnosis, genome-wide sequencing should be considered early in the diagnostic workup.

At the time of diagnosis, clinicians should ensure a comprehensive baseline assessment, including neurological evaluation with electroencephalography when seizures are present or suspected, brain MRI to document structural findings, and feeding assessment in individuals with dysphagia or aspiration risk [2,9-10]. Respiratory evaluation should be considered in those with a history of neonatal hypoventilation, recurrent respiratory infections, or suspected sleep-disordered breathing [9]. Phenotype-guided screening for cardiac, endocrine, and urogenital abnormalities may also be appropriate, particularly in individuals with chromosomal deletions involving 5q31.3 [18,40].

Neurological follow-up should focus on seizure monitoring, developmental progress, and detection of movement disorders. Antiseizure medication regimens should be regularly reviewed for efficacy and tolerability. In individuals with refractory epilepsy, referral to specialized epilepsy centers may be warranted to consider dietary therapy or device-based interventions [19,52]. Developmental regression or new neurological symptoms should prompt reassessment.

Early and sustained rehabilitation services are critical. Physical therapy and occupational therapy should begin in infancy to support motor development, prevent contractures, and optimize functional mobility [49-50]. Assistive devices, orthotics, and adaptive equipment may enhance independence and safety. Speech therapy is essential, and augmentative or alternative communication systems should be introduced early in individuals with severe expressive language impairment [9].

Feeding and nutritional management require close monitoring, particularly during infancy and periods of rapid growth. Swallowing dysfunction and gastroesophageal reflux should be actively addressed to reduce aspiration risk and ensure adequate caloric intake [5,24]. Gastrostomy tube placement may be appropriate in cases of persistent feeding difficulty or failure to thrive [41-42]. Regular growth assessments and nutritional counseling are recommended.

Orthopedic surveillance should include periodic assessment for scoliosis, hip instability, and contractures, particularly during childhood and adolescence when musculoskeletal complications may progress [12-13]. Early identification allows for conservative measures such as bracing, with surgical referral considered when indicated.

Respiratory care should emphasize prevention and early management of infections, vaccination adherence, and evaluation for sleep apnea when clinically suspected [2,9]. In individuals requiring surgical procedures, preoperative planning should account for potential airway management challenges and increased sensitivity to respiratory depressant medications [51].

Genetic counseling is an essential component of care. Families should be informed of recurrence risk, the possibility of germline mosaicism, and the availability of prenatal or preimplantation genetic testing when appropriate [11,18]. Ongoing psychosocial support and connection to patient advocacy organizations may assist families in navigating long-term care needs.

Transition planning during adolescence should address continuity of medical care, educational and vocational support, and coordination between pediatric and adult services. Because long-term natural history data remain limited, continued surveillance into adulthood is recommended.

Increasing awareness of PURA-NDD among healthcare professionals is essential for improving diagnostic recognition and patient outcomes. Because the early manifestations, such as hypotonia, feeding difficulties, and developmental delay, overlap with many other pediatric conditions, PURA-NDD may remain underrecognized without increased clinical awareness and a high index of suspicion. Broader familiarity with the characteristic clinical constellation and greater access to genomic sequencing can facilitate earlier diagnosis, allowing timely initiation of multidisciplinary management, appropriate genetic counseling, and enrollment in patient registries or research initiatives. Educational efforts directed toward pediatricians, neurologists, neonatologists, and clinical geneticists may therefore play an important role in improving the detection of this rare disorder.

In summary, clinical care in PURA-NDD should be anticipatory, multidisciplinary, and individualized. Regular reassessment across neurological, respiratory, nutritional, musculoskeletal, and developmental domains can mitigate secondary complications and improve overall quality of life.

## Conclusions

PURA-NDD is an ultra-rare genetic condition characterized by neonatal hypotonia, severe expressive language impairment, intellectual disability, epilepsy, and multisystem involvement. Advances in genomic sequencing have improved diagnostic recognition and enabled earlier multidisciplinary intervention. Current management remains supportive and symptom-directed, focusing on seizure control, respiratory and nutritional support, rehabilitation, and surveillance for orthopedic and systemic complications.

Although no disease-modifying therapy is currently available, expanding insights into PURA haploinsufficiency and neuronal RNA regulation have established a biologically coherent foundation for future therapeutic development. Continued international collaboration, natural history studies, and patient-centered research infrastructure will be essential to advance trial readiness and translate emerging molecular strategies into meaningful clinical interventions.
